# Fibrillar Nanomembranes of Recombinant Spider Silk Protein Support Cell Co-culture in an *In Vitro* Blood Vessel Wall Model

**DOI:** 10.1021/acsbiomaterials.1c00612

**Published:** 2021-06-25

**Authors:** Christos Panagiotis Tasiopoulos, Linnea Gustafsson, Wouter van der Wijngaart, My Hedhammar

**Affiliations:** † School of Engineering Sciences in Chemistry, Biotechnology, and Health, Department of Protein Science, AlbaNova University Center, KTH—Royal Institute of Technology, Roslagstullsbacken 21, 114 21 Stockholm, Sweden; ‡ School of Electrical Engineering and Computer Science, Division of Micro and Nanosystems, KTH—Royal Institute of Technology, Malvinas väg 10, 114 28 Stockholm, Sweden

**Keywords:** basement membrane, cell co-culture, nanomembrane, recombinant spider silk, tissue engineering, vessel wall

## Abstract

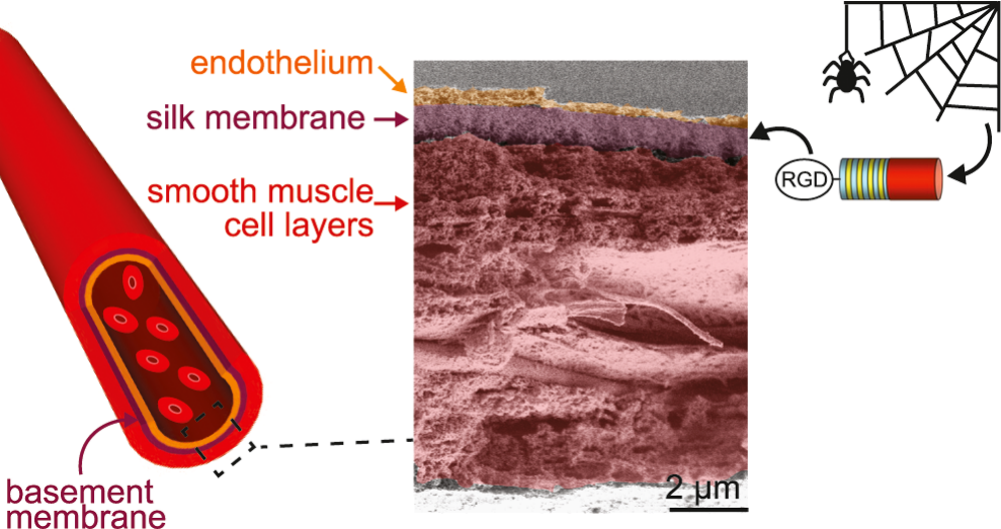

Basement membrane is a thin but dense network of self-assembled extracellular matrix (ECM) protein fibrils that anchors and physically separates epithelial/endothelial cells from the underlying connective tissue. Current replicas of the basement membrane utilize either synthetic or biological polymers but have not yet recapitulated its geometric and functional complexity highly enough to yield representative *in vitro* co-culture tissue models. In an attempt to model the vessel wall, we seeded endothelial and smooth muscle cells on either side of 470 ± 110 nm thin, mechanically robust, and nanofibrillar membranes of recombinant spider silk protein. On the apical side, a confluent endothelium formed within 4 days, with the ability to regulate the permeation of representative molecules (3 and 10 kDa dextran and IgG). On the basolateral side, smooth muscle cells produced a thicker ECM with enhanced barrier properties compared to conventional tissue culture inserts. The membranes withstood 520 ± 80 Pa pressure difference, which is of the same magnitude as capillary blood pressure *in vivo*. This use of protein nanomembranes with relevant properties for co-culture opens up for developing advanced *in vitro* tissue models for drug screening and potent substrates in organ-on-a-chip systems.

## Introduction

*In vitro* biological systems with high mimicry to *in vivo* conditions are in great demand, as they can alleviate the burden from heavy animal use and facilitate personalized treatment by using patients’ own cells. Many such systems employ porous membranes that aim to mimic the basement membrane of various tissues. Porous membranes that separate epithelial/endothelial cells from the cells of the underlying connective tissue can emulate the complex microenvironments of brain, retina, lung, and blood vessels *ex vivo*.^1−5^ In addition, the co-culture of cells onto such membranes has been followed to study complex biological functions, including cell–cell communication, cell–matrix interaction, and barrier formation.^6−8^

The basement membrane is a thin (the thickness varies with the tissue type) but dense network of self-assembled extracellular matrix (ECM) protein fibrils, mainly laminin and collagen type IV, which surrounds and separates most tissues and structurally supports cells. The membrane acts as a signaling platform by its ability to tether several growth factors, that is, vascular endothelial growth factor, transforming growth factor-β, and fibroblast growth factor, through binding interactions between its different elemental components.^9^ Hence, it is involved in many cell signaling events, such as cell survival, proliferation, and polarization.

The gold standard to a basement membrane mimic is still the use of commercial tissue culture inserts (TC-inserts). TC-inserts are manufactured by track-etching nanopores in inert polymer membranes [*i.e.*, polyethylene terephthalate (PET)]. However, the membrane thickness (>10 μm), rigidity, chemical nature, and nanoscale structure of such TC-inserts do not resemble those of the membranes in native tissues.^10−12^ Thus, alternative materials and fabrication techniques have been investigated to generate replicas that better imitate the basement membranes. Several synthetic materials, that is, polydimethylsiloxane,^13^ polytetrafluoroethylene (PTFE),^14^ PET,^15^ silicon carbide,^16^ or silicon dioxide,^10^ and biopolymers, such as collagen, alginate, Matrigel, and composites thereof,^17−21^ have been utilized. Synthetic polymers feature excellent fabrication properties and robustness but are not biodegradable. The basement membrane, in contrast, is a dynamic environment that the cells constantly remodel to sustain specific cell functions.^22^ Further, several synthetic polymers (*i.e.*, PTFE) have extremely low surface energy and need to be coated with ECM proteins to facilitate cell attachment.^23^ By using biological polymers instead, it is challenging to construct nanomembranes (*i.e.*, membranes less than 1 μm thick) that are uniform across the entire surface.^21^ Moreover, batch-to-batch variation and partially defined ECM composition are the disadvantages associated with the use of, for example, Matrigel in cell culture applications.^24−26^

As an alternative membrane fabrication technique, electrospinning can be used to cast materials into fibrillar biomimetic matrices that promote cell attachment and efficiently direct migration and differentiation.^27,28^ Electron beam lithography, followed by plasma etching can be used to form thin and flexible nanoporous membranes more suitable for cell culture applications.^11^ Evaporation-driven techniques using ECM proteins have yielded considerably thinner membranes that were also shown to better regulate the permeation of molecules than track-etched membranes.^20,21^ Despite significant progress, the vast majority of current membrane replicas fail to fully recapitulate the complexity of natural basement membranes in at least one aspect (Table S1, Supporting Information).

**Figure 1:**
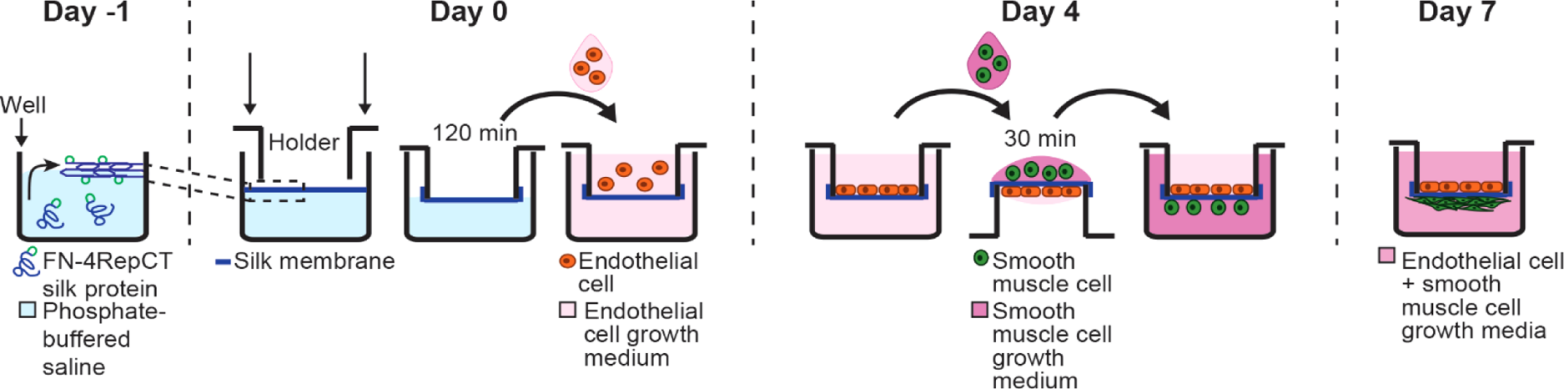
Overview of the procedure for the preparation of and cell seeding on silk membranes. Day −1: a solution of FN-4RepCT silk protein diluted in phosphate-buffered saline (PBS) is placed in an open well where the protein self-assembles into a membrane at the air–liquid interface overnight. Day 0: a holder is lowered onto the membrane, which adheres to the holder over 2 h. The holder with the membrane is lifted from the interface and placed in endothelial cell growth medium, and human dermal microvascular endothelial cells (HDMEC) are seeded on the apical side of the membrane. Day 4: the holder is reversed, smooth muscle cells (SMC) are seeded on the basolateral side of the membrane, and allowed to adhere for 30 min, after which the holder is placed in SMC growth medium and filled with endothelial cell growth medium. Day 7: HDMEC have established a confluent monolayer on the apical side, and SMC have produced a thick ECM on the basolateral side of the silk membrane. Drawing is not in scale. FN-4RepCT silk: 4RepCT silk protein functionalized with the Arg-Gly-Asp (RGD)-containing cell-binding motif from fibronectin.

A promising material for constructing basement membrane replicas is recombinant spider silk protein, as it forms structures that are strong and elastic,^29^ biocompatible, and biodegradable.^30,31^ Further, the silk protein self-assembles, under mild conditions, into nanofibrillar structures similar in morphology to the ECM.^32^ Recombinant spider silk proteins have previously been used to fabricate thick (3–9 μm), nonporous membranes to model the retinal pigment epithelium.^33^ Yet, recombinant spider silk proteins have recently been shown to form thinner (250 nm) and bioactive membranes that are permeable to human plasma proteins, are mechanically robust, and support the formation of confluent monolayers of epidermal skin cells (keratinocytes).^34^ Herein, we report on recombinant spider silk nanomembranes able to support a cell co-culture into an *in vitro* blood vessel wall model (Figure 1).

## Materials and Methods

### Cell Cultures

HDMEC isolated from the dermis of juvenile foreskin and adult skin (PromoCell, Heidelberg, Germany) were cultured and expanded in endothelial cell growth medium MV2 ready to use (PromoCell, Heidelberg, Germany) supplemented with 1% antimycotics/antibiotics. Primary human SMC isolated from coronary artery (Thermo Fisher Scientific, Waltham, MA, USA) were cultured and expanded in complete SMC growth medium (Gibco, Waltham, MA, USA) supplemented with 5% fetal bovine serum and 1% penicillin–streptomycin. HDMEC and SMC were used at passage 7. The growth medium in all cell cultures was changed every second day.

### Preparation of Silk Membranes

The 4RepCT silk protein functionalized with the RGD-containing cell binding motif from fibronectin (FN-4RepCT) (Spiber Technologies AB, Stockholm, Sweden) was thawed at room temperature and spun down for a minute using a bench-top centrifuge. The silk protein was diluted in PBS (pH 7.4) (National Veterinary Institute, Uppsala, Sweden) to a final concentration of 0.5 mg/mL and subsequently added into wells of 24-well polystyrene plates with a hydrophobic surface (Sarstedt, Nümbrect, Germany). The prepared silk solution self-assembled into membranes, under sterile conditions, at the air–liquid interface of the wells at room temperature overnight. A custom-made 3D-printed holder from polylactic acid (NatureWorks LLC, Minnetonka, MN, USA) was lowered onto the membrane and allowed to sit for 2 h during which the membrane sealed around the holder. After this, the membrane could be lifted from the interface.

### Cell Seeding of Silk Membranes

The full process is illustrated in Figure 1. HDMEC were harvested when reaching about 85% confluency according to commonly followed protocols. The cells were washed once with PBS and enzymatically detached with TrypLE Express (Life Technologies, Waltham, MA, USA) to be prepared to a 10^6^ cells/mL solution. The silk membranes were lifted from the interface as described above and transferred into wells of a tissue-culture-treated 24-well plate (Corning Inc., New York, NY, USA) that contained MV2 medium below and above the membranes. HDMEC were seeded onto silk membranes, as well as TC-inserts (Sarstedt, Nümbrect, Germany), in a final density of 0.25 × 10^6^ cells per 20 μL per membrane. Nonadherent cells and cell debris were removed the next day with culture medium change.

On day 4, SMC were prepared as described above and seeded onto the opposite side of silk membranes and TC-inserts, in a final density of 0.15 × 10^6^ cells per 20 μL per membrane. SMC were allowed to adhere to the membranes at 37 °C with 5% CO_2_ and 95% humidity for 30 min and then transferred back to the wells that contained SMC and MV2 growth media below and above the membranes, respectively.

### Transendothelial Electrical Resistance

On days 1, 3, 5, and 7, the transendothelial electrical resistance (TEER) was measured at 6 V and 0.22 A on silk membranes and TC-inserts (*n* = 6) using an epithelial voltohmmeter (EVOM^2^) (World Precision Instruments, Sarasota, FL, USA). TEER was also measured on silk membranes and TC-inserts that did not contain cells (*n* = 3). On days 5, 6, and 7, TEER was measured on silk membranes with only SMC (*n* = 4). The average value of the membranes without cells was subtracted from each value measured on the membranes with cells. Final values are expressed in Ω·cm^2^ based on the respective cell growth area for silk membranes and TC-inserts.

### Permeation Studies

On days 4 and 7, the permeation of molecules of various sizes was studied on silk membranes and TC-inserts without cells, seeded with only HDMEC, seeded with only SMC (only silk membranes), and double-seeded. Dextran (Thermo Fisher Scientific, Waltham, MA, USA) of 3 or 10 kDa was combined with IgG (Thermo Fisher Scientific, Waltham, MA, USA) and 3 μm diameter fluorescein isothiocyanate (FITC)-labeled beads (Sigma-Aldrich, St. Louis, MO, USA). 300 μL of the prepared mixture was loaded above the silk membranes and TC-inserts (*n* = 6). After 1 h of incubation at 37 °C with 5% CO_2_ and 95% humidity, part of the growth medium was collected from below the membranes and TC-inserts, and the fluorescence intensity was measured using a plate reader (CLARIOstar, BMG Labtech, Ortenberg, Germany). The intactness of silk membranes was verified using an inverted fluorescence microscope (Nikon Eclipse Ti, Tokyo, Japan), where the presence of 3 μm FITC-labeled beads indicated leakage. These membranes were excluded from the analysis. Leakage through silk membranes was visualized by adding dissolved patent blue (Sigma-Aldrich, St. Louis, MO, USA) on top of the membrane.

### Mechanical Studies

On days 4 and 7, the mechanical properties of silk membranes (*n* > 5), both with and without cells, were evaluated using a standard bulging experiment. On day 0, only the mechanical properties of silk membranes without cells (*n* = 5) were evaluated. In short, the holder with the membrane was inverted and positioned on a hollow cylindrical holder inside a large beaker. Water was slowly added outside the cylinder. The inflation of the membrane and pressure difference outside and inside the cylinder was recorded using a standard camera (Canon EOS 600D). The image right before bursting was extracted using MATLAB (R2020a). Pixel counting was used to determine the height (Δ*h*) of the inflated membrane as well as the pressure difference (Δ*P*).

### Sample Preparation for Scanning Electron Microscopy

Cells on the silk membranes and TC-inserts were fixed in 2% glutaraldehyde (Sigma-Aldrich, St. Louis, MO, USA) in 0.1 M *N*-(2-hydroxyethyl)piperazine-*N*′-ethanesulfonic acid (HEPES) buffer. The fixed cells were washed three times with 0.1 M HEPES buffer for 5 min each, before being serially dehydrated with 50% ethanol (two times, for 10 min each), 70% ethanol (two times, for 10 min each), 95% ethanol (two times, for 10 min each), and 99.5% ethanol (three times, for 15 min each) on an agitation shaker. Hexamethyldisilazane (HMDS, Sigma-Aldrich, St. Louis, MO, USA) was then used to serially dry the fixed samples for 15 min with two parts 99.5% ethanol and one part HMDS, 15 min with one part 99.5% ethanol and one part HMDS, 15 min with one part 99.5% ethanol and two parts HMDS, and finally 15 min with HMDS alone for three times. The last HMDS was let to evaporate overnight under a fume hood, and the samples were then mounted on a conductive carbon tape, sputter-coated with a 12 nm thick layer of gold, and images were acquired using a scanning electron microscope (Zeiss, Oberkochen, Germany). The thickness of the membranes (*n* = 6) and produced ECM was measured using pixel counting in MATLAB. The measured ECM thickness was divided by the average thickness of the membranes to eliminate the effect of any tilt in the images.

### Cell Fixation and Immunostaining

HDMEC and SMC were washed twice with prewarmed PBS and fixed in 4% paraformaldehyde for 10 min at room temperature. The cells were then washed twice with PBS, permeabilized with 0.2% Triton X-100 in PBS for 10 min, washed twice with 0.05% Tween in PBS for 5 min, and finally, blocked with 1% goat serum (GS) in PBS with 0.05% Tween for 60 min. Primary antibodies against the proteins of interest were diluted according to the recommended dilution factors in 1% GS in PBS with 0.05% Tween and allowed to incubate overnight onto the membranes at +4 °C. A primary antibody inventory and the used dilution factors are listed in Table S2 (Supporting Information). The cells were subsequently washed twice with 0.05% Tween in PBS for 5 min, and the respective secondary antibodies diluted in 1% GS in PBS with 0.05% Tween were added and allowed to incubate for 2 h at room temperature. Nuclear staining was performed with DAPI for 10 min. The stained cells were washed twice with 0.05% Tween in PBS for 5 min, mounted on microscopic glasses using Dako fluorescence mounting medium (Dako North America, Carpentaria, CA, USA), and documented using fluorescence microscopy (Nikon Eclipse Ti, Tokyo, Japan). Images were captured using the NIS Elements BR software, and blurriness was subtracted using the Unsharp Mask command (radius 2.0 pixels and mask weight 0.60) on ImageJ.

### Statistics

Statistical analysis was performed in Microsoft excel (16.44) using the data analysis *T* test tool. Statistical significance was considered as *p < 0.05, **p < 0.01, and ***p < 0.001.

## Results and Discussion

### Silk Membrane Characterization

Spider silk membranes were formed by allowing the solutions of silk proteins in open wells to stand still at ambient conditions overnight. During this time, the silk proteins self-assemble at the air–liquid interface. The structural rearrangement of silk proteins to form a nanofibrillar membrane corresponds to a continuous reduction of α-helices in favor of increased β-sheet conformations.^34^ The content of β-sheet formation has previously been reported to account for the extensibility of silk proteins,^35^ as well as the unfolding and elasticity of, for example, fibrin.^36^ The membranes can be lifted from the interface by lowering the custom-made 3D-printed holder (Figure S1) and allowing the membrane to detach from the walls of the well and instead adhere to the holder. The thickness of the membrane can be altered by varying the silk concentration or the assembly time.^34^ Noteworthily, the thickness of the silk membranes increases over time, from 470 ± 110 nm at day 0 to 690 ± 150 nm at day 7, by keeping them submerged in cell culture media (Figure S2). The serum and growth factor components in the media adsorb onto each side of the silk membranes, thereby adding particular bioactive properties. Such bioactivation opens up for cell culture applications, wherein the surfaces need to be coated with specific proteins that facilitate cell attachment, proliferation, and growth.

**Figure 2:**
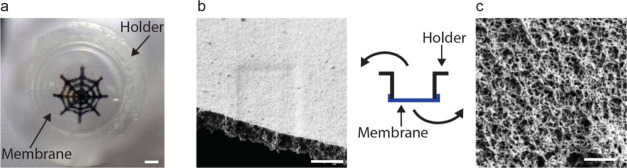
Appearance of the spider silk membrane. (a) Photograph of a spider web illustration as seen through the silk membrane, showing its optical transparency. Scale bar = 1 mm. (b) Tilted SEM image of the smooth air-side (apical) and cross section. (c) SEM image of the textured liquid-side (basolateral). Scale bars = 1 μm.

The silk nanomembranes have several other properties that make them suitable for emulating the basement membrane *in vitro*. Beyond their nanoscale thickness and internal fibrillar structure, they are permeable to proteins^34^ and are optically transparent (Figure 2a). The latter is important for microscopy and further analysis.^20^ Noteworthily, the two sides of the membrane have different appearances; while the side facing air during formation (from here on the air-side) is smooth, the side facing the solution during formation (from here on the liquid-side) is textured from silk aggregates (Figure 2b–c). Despite their difference in appearance, both sides support cell attachment and growth,^34^ thereby making them suitable for co-culture applications.

### Establishment of Endothelium and Production of ECM on Silk Membranes

**Figure 3:**
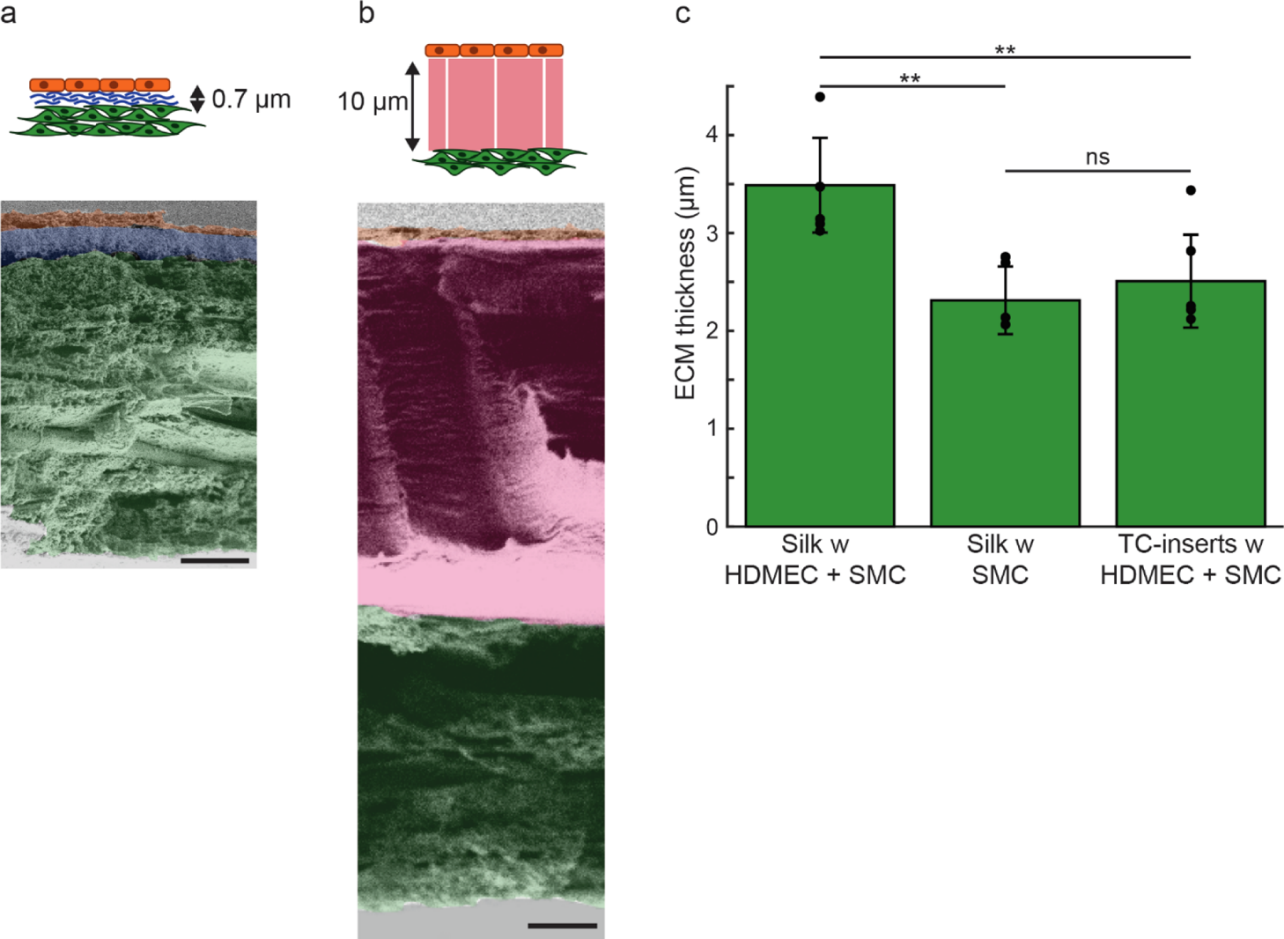
ECM produced by SMC. (a) Sketch (not drawn to scale) of the silk membrane (in blue) seeded with endothelial cells (HDMEC) on the apical side (in orange) and SMC on the basolateral side and the ECM produced by the latter (in green), with the representative SEM image which has been false-colored to match the sketch. (b) Sketch (not drawn to scale) of a TC-insert (in pink) seeded with both HDMEC (in orange) and SMC and the ECM produced by the latter (in green), with the representative SEM image which has been false-colored to match the sketch. Scale bars = 2 μm. (c) Measured thickness (mean ± SD) of ECM for silk membranes and TC-inserts seeded with both HDMEC and SMC, as well as silk membranes seeded with only SMC. **P < 0.01, ns—not significant (*P* > 0.05). HDMEC: human dermal microvascular endothelial cells.

After formation, the silk membranes were seeded with endothelial cells (HDMEC) on the air-side and kept in culture for 4 and 7 days. Within this time frame, the cells adhered, stretched, flattened out (Figure S3a), and formed a confluent monolayer (Figure S3b) of 540 ± 310 nm in thickness (Figure S3c). This thickness is within the range of what has been reported for the endothelium lining of blood vessels *in vivo*.^37^ Unlike previously reported weak cell attachment^38^ and poor spreading^39^ to silk matrices, the silk membranes produced herein promoted a firm adhesion and homogeneous cell spreading across the entire surface area. The fast establishment of a confluent endothelium is likely attributed to the RGD-containing cell binding motif fused with the silk protein at the gene level to facilitate cell attachment and proliferation.^40^ The self-assembly process does not appear to have affected the exposure of the RGD motif on the surface, thereby allowing the development of integrin-mediated cell attachment. Integrins are glycoproteins highly expressed by vascular endothelial cells with strong affinity to peptide sequences containing the RGD motif.^41^ Besides cell adhesion, integrins are also involved in cell proliferation, migration, differentiation, and growth. Noteworthily, the silk membranes were stable enough for the handling and seeding of cells, although only submicrometer-thin; thus less than a tenth the thickness of the track-etched membranes in TC-inserts that was used as control (Figure 3a–b).

On day 4, SMC were seeded on the liquid-side of silk membranes and TC-inserts and kept in culture medium until day 7. Silk membranes that did not contain HDMEC on the air-side were also seeded with SMC. To confirm the phenotype, SMC were stained for alpha-smooth muscle actin on double-seeded silk membranes as well as on silk membranes without HDMEC. Positive signal was detected on both conditions (Figure S4a) but also on silk membranes that were only seeded with HDMEC (Figure S4b). The ECM produced by the SMC on the double-seeded silk membranes was significantly thicker than that on the silk membranes without HDMEC as well as double-seeded TC-inserts (*p* < 0.01) (Figure 3c). Noteworthily, SMC on double-seeded TC-inserts produced an equally thick ECM (*p* > 0.05) to silk membranes without HDMEC on the air-side. The ability of SMC on double-seeded silk membranes to synthesize a protein-rich ECM is most likely due to the response to signaling molecules by HDMEC, indicating the establishment of communication between them. In contrast, the thicker (10 μm) and nanoporous PET membrane of TC-inserts may have less efficiently facilitated the diffusion of soluble factors across the juxtaposed cells, which is fundamental in the blood vessel wall.^42^ In a previously reported cell co-culture study, wherein a 13 μm thick and porous PET membrane was employed, SMC were found to develop cytoplasmic projections that traversed through the pores of the membrane and made contact with the endothelial cells.^43^ However, approximately 20% of the pores were blocked by the cytoplasmic projections. Hence, the diffusion of signaling molecules across the porous membranes of TC-inserts may greatly be affected. In contrast, the fibrillar nature of silk membranes resembles better the morphology of the basement membrane and does not pose such an issue. Previous studies have also demonstrated the importance of a biomimetic nanofibrous substrate for cells to adhere more strongly and thereby induce the production of ECM.^44,45^ Further, close proximity between endothelial cells and SMC is of great importance for the regulation of vascular tone, by accordingly tuning the properties of the ECM with protein synthesis, both in healthy and diseased vessels.^46^

Immunofluorescence staining revealed the presence of key ECM components (collagen types I and III, elastin, and hyaluronic acid) secreted by the cells on double-seeded silk membranes (Figure S5a–h). The deposition of fibrillar structures, most probably collagen fibrils, on the air-side was also confirmed by SEM (Figure S5i–l). Besides collagen and elastin, the presence of hyaluronic acid is also of great importance as it is involved in the dimensional stabilization of ECM, via noncovalent interactions, as well as in the stability of glycocalyx, a glycoprotein on the luminal surface of endothelial cells regulating the permeability and vascular tone.^47^

### Mechanical Properties of Silk Membranes

**Figure 4:**
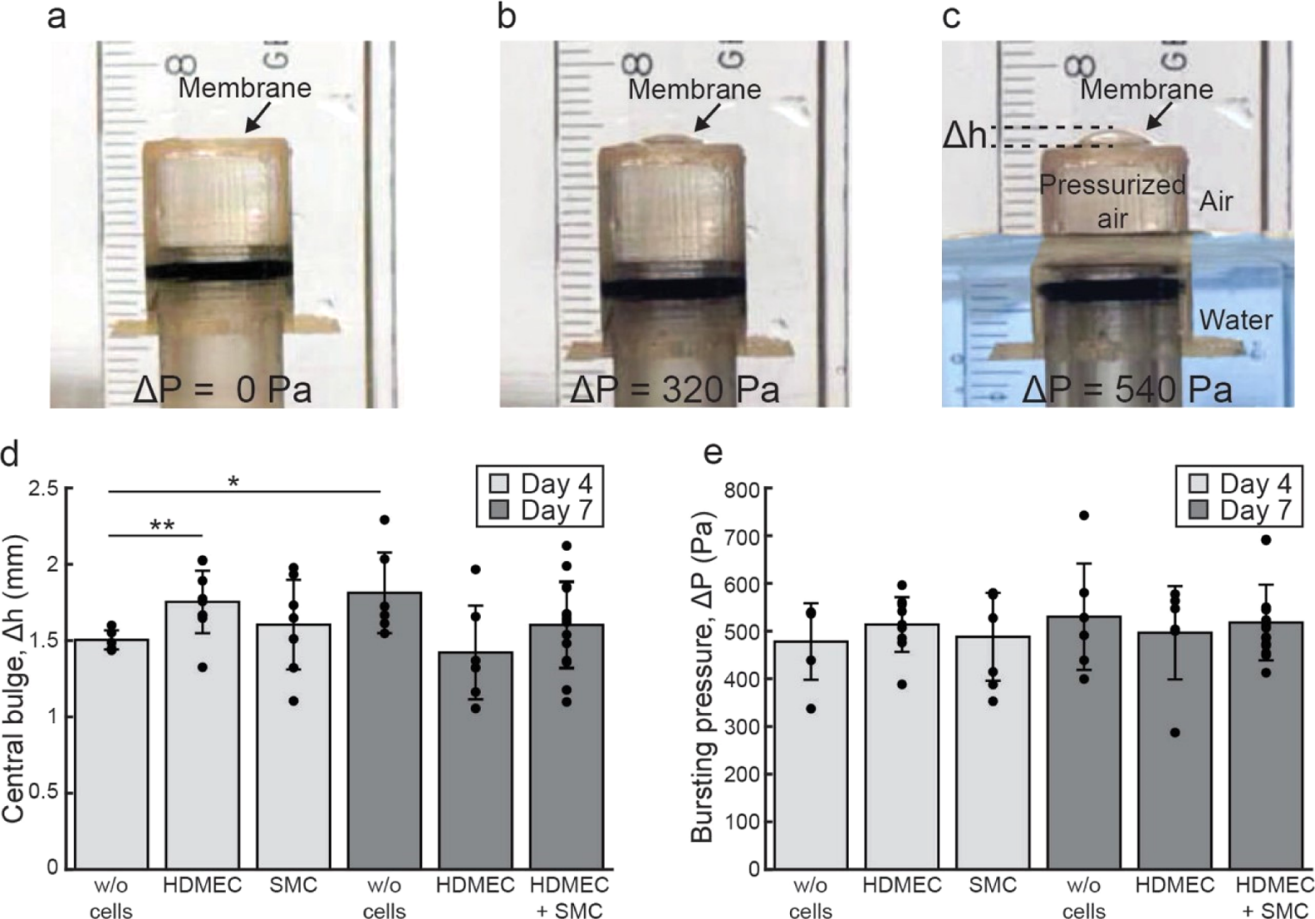
Mechanical properties of spider silk membranes with and without cells. Photographs of a bulging membrane under pressure differences (Δ*P*) of (a) 0, (b) 320, and (c) 540 Pa. The air pressure inside the holder is regulated hydrostatically using a water column, visible only in (c) (false-colored blue). Ruler is mm-scaled. Plots showing the (d) average center deflection (Δ*h*) (mean ± SD) of the membrane and the corresponding (e) pressure difference (Δ*P*) (mean ± SD) at burst for silk membranes without cells, with endothelial cells (HDMEC), with SMC, and both cell types (HDMEC + SMC) after 4 (light gray) and 7 (dark gray) days in culture. **P < 0.01, *P < 0.05. HDMEC: human dermal microvascular endothelial cells.

The mechanical properties of spider silk membranes with and without cells were characterized using a standard bulging experiment. Briefly, the holder with the membrane was inverted and attached to a cylindrical stand. The setup was placed in a beaker, and water was slowly added to the outside of the cylinder, generating a hydrostatic pressure in the air column inside the stand (Figure S6a). Thereby, the pressure difference caused the membrane to bulge until burst (Figures 4a–c and S6b–e). The process was performed for silk membranes without cells on days 0, 4, and 7, as well as for single- and double-seeded silk membranes on days 4 and 7. Silk membranes without cells on day 7 bulged slightly more than the respective membranes on days 0 (1.4 ± 0.2 mm, *p* < 0.05) and 4 (1.5 ± 0.1 mm, *p* < 0.05) (Figures 4d and S7a). All silk membranes with cells exhibited similar bulging profiles, indicating that the cells and the ECM deposited by them are well adapted to the silk membrane properties. Thus, fully stretchable cell-seeded silk membranes were obtained, which is a prerequisite in mimicking the blood vessel wall.

No significant difference in burst pressure between the membranes without cells was observed, except for silk membranes on day 0 that burst at a lower pressure (220 ± 80 Pa, *p* < 0.05) (Figure S7b). We therefore assume that serum and growth factor components adsorbed from the media not only increase the thickness but also enhance the mechanical properties of the silk membranes. Further, all membranes past day 4 could withstand pressures of approximately 500 Pa (Figure 4e), which is of the same magnitude as capillary blood pressure *in vivo* (1300–3000 Pa).^48^ It should be noted though that our measurement setup is limited to one direction bulge, in contrast to the bidirectional blood pressure applied against vessel walls during systole/diastole *in vivo*. To address the issue of bidirectionality, the silk membranes can in future be incorporated in a microfluidic chip, wherein the growth medium can be circulated. Hence, shear stresses applied upon the endothelium may generate stronger membranes withstanding higher pressures. However, the pressure at burst found herein is in line with other reported pressures typically applied on synthetic membranes^5^ as well as collagen gels^49^ in biomimetic microfluidic blood vessel models. Thus, the silk membranes may also be considered for tissue models, wherein contraction/compression forces are exerted onto cells, that is, in the heart and lungs.

### Barrier Properties of Silk Membranes

**Figure 5:**
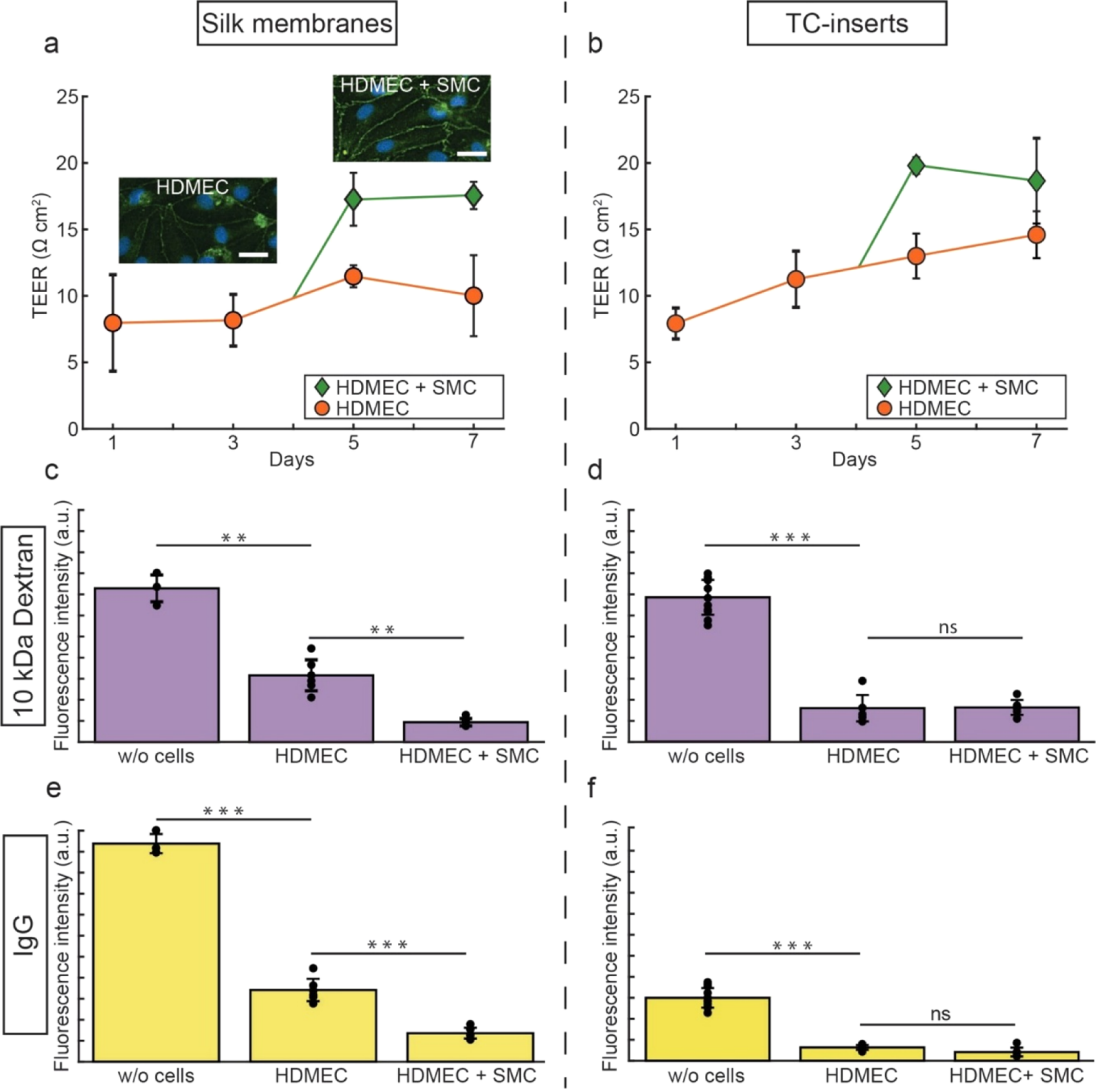
Barrier properties of silk membranes and TC-inserts with and without cells. Normalized TEER values (mean ± SD) for (a) silk membranes and (b) TC-inserts seeded with only endothelial cells (HDMEC) and with both HDMEC and SMC (HDMEC + SMC) at different days in culture. Inserted micrographs in (a) show HDMEC stained for tight junctions (zona occludens-1, in green) and cell nuclei (DAPI, in blue) on days 4 and 7. Scale bars = 25 μm. Permeation of 10 kDa dextran (mean ± SD) through (c) silk membranes and (d) TC-inserts without cells, seeded with HDMEC, and both cell types (HDMEC + SMC). Permeation of IgG (mean ± SD) through (e) silk membranes and (f) TC-inserts for the same conditions, all after 7 days in culture. ***P < 0.001, **P < 0.01, ns—not significant (*P* > 0.05). HDMEC: human dermal microvascular endothelial cells.

The barrier properties developed by cells on silk membranes and TC-inserts were investigated by TEER and permeability measurements. The electrical resistance was measured every second day, and barrier integrity was confirmed until the last day of culture (Figure 5a–b). HDMEC on silk membranes stained for zona occludens-1 revealed the formation of tight junctions both before and after the addition of SMC on the liquid-side (Figure 5a). Enhanced barrier properties were noticed for double-seeded silk membranes, in contrast to silk membranes that contained either only HDMEC or SMC (Figure S8). This difference in barrier tightness likely results from the development of cell communication between HDMEC and SMC that were in close proximity. The results are in line with previously reported studies wherein a co-culture of cells generated a tighter barrier as compared to monoculture.^50,51^ No significant difference was observed between double-seeded silk membranes and TC-inserts.

To determine the permeation capacity of the membranes, their apical side was loaded with fluorescent-labeled dextran (3 and 10 kDa), as compounds indicative of paracellular permeation (<70 kDa^52^), and IgG (150 kDa), as a typical example of transcellular permeation. Physical intactness of the membranes was confirmed using fluorescent-labeled microbeads (Figure S9), previously demonstrated not to permeate intact silk membranes.^34^ All molecules loaded on the air-side permeated through silk membranes with or without cells (Figures 5c–f and S10). As expected, silk membranes and TC-inserts without cells allowed significantly more permeation compared to those with cells. Interestingly, on day 7 and for all cell culture combinations, silk membranes allowed significantly more permeation of IgG and 3 kDa dextran as compared to TC-inserts under similar conditions. For 10 kDa dextran, no significant difference was observed. Recent findings indicate that permeation is hindered on transwell membranes with similar pore size as in the TC-inserts used herein (0.4 μm).^12^ Besides pore size, the membrane material as well as charge, hydrophilicity, and shape of the loaded molecule may also influence permeation, which could explain the differences observed between 3 and 10 kDa dextran.

## Conclusions

Optimally tuning biomaterials to match the specific features of the basement membrane remains challenging, and the material that completely combines all aspects is yet to be found. Herein, the inherent properties of recombinant spider silk protein to self-assemble under very mild conditions at interfaces resulted in the formation of a membrane similar in morphology to the basement membrane. Although exceeding conventional TC-inserts as *in vitro* blood vessel wall mimics, the formed silk membranes are simplified versions, matching better the basement membrane thickness of bigger vessels (*i.e.*, aorta) than that of peripheral vasculature. However, by simply altering the silk concentration, the thickness can easily be adjusted to equal that of basement membranes in smaller vessels. Future studies are therefore needed to investigate the ability of thinner (<500 nm) silk membranes to support a cell co-culture. Further, the pressure that the silk membranes can withstand was found to be in the lower range of the blood pressure applied *in vivo*. Yet, *in vivo* cells are constantly under stresses, whereas static cultures were examined herein. Future work should thus focus on subjecting silk-based tissue models to shear stresses (*e.g.,* in a microfluidic chip) and expose endothelial cells to native-like conditions. As such, we anticipate that not only the mechanical properties may be improved but also the permeation to molecules will be affected (by the formation of tighter junctions), thereby resulting in even more *in vivo*-like basement membrane replicas.

To summarize, this study demonstrated that the silk membranes feature a combination of unique properties, that is, nanoscale thickness, millimeter-sized diameter, internal fibrillar structure, and flexibility yet sturdiness, to lead to improved basement membrane replicas. As such, we anticipate that our silk membranes would be of great use as substrates in systems for *in vitro* drug screening and in organs-on-a-chip.

## Supporting Information Available

Limited overview of previous studies on membrane mimics; inventory of primary antibodies used in this study; photograph of tissue culture inserts; thickness of silk membranes without cells; SEM images and bar plot of the formed endothelium on silk membranes; immunofluorescence images confirming the α-SMA phenotype; immunofluorescence and SEM images of key ECM proteins; photographs of the setup used for bulging experiments; bar plots for the mechanical properties of silk membranes without cells; TEER values for double- and single-seeded silk membranes; photographs and fluorescence images on intact and leaky silk membranes; and bar plots of permeability to 3 and 10 kDa dextran and IgG for silk membranes and TC-inserts (PDF)
