# Supplementary material for: Fibrillar Nanomembranes of Recombinant Spider Silk Protein Support Cell Co-culture in an In Vitro Blood Vessel Wall Model
Source: ACS Biomater Sci Eng. 2021 Jun 25;7(7):3332–9. doi: 10.1021/acsbiomaterials.1c00612 (PMC8290846; doi:10.1021/acsbiomaterials.1c00612)
Supplement: Supplementary file 1 — ab1c00612_si_001.pdf [file ab1c00612_si_001.pdf]

Supporting information

Fibrillar nanomembranes of recombinant spider silk protein support cell co-culture in an *in vitro* blood vessel wall model

Christos Panagiotis Tasiopoulos[1], Linnea Gustafsson[2], Wouter van der Wijngaart[2], and My Hedhammar[1*]*

[1] KTH – Royal Institute of Technology, School of Engineering Sciences in Chemistry, Biotechnology, and Health, Department of Protein Science, AlbaNova University Center, Roslagstullsbacken 21, 114 21 Stockholm, Sweden

[2] KTH – Royal Institute of Technology, School of Electrical Engineering and Computer Science, Division of Micro and Nanosystems, Malvinas väg 10, 114 28 Stockholm, Sweden

***Corresponding authors**

Wouter van der Wijngaart, email: wouter@kth.se

My Hedhammar, email: myh@kth.se

Table S1. A limited overview of membrane mimics used to model various tissues or as substrates for cell culture applications. The membranes are classified by the material used to fabricate them as synthetic and biologic polymers. Other properties of interest are: the thickness, fibrillar structure, biodegradability, permeability, and intended application of the engineered membranes.

Material	Conformation	Thickness	Fibrillar	Biodegradable	Permeability	Application	Reference
PET	Porous membrane	23 µm	No	No	Sodium fluorescein (376 Da), fluorescein isothiocyanate -labeled dextran (4.4 kDa), and Evans blue-labeled albumin (67 kDa)	Intestinal and lung epithelial barriers, BBB	[1]
Polyester	Porous membrane	20 µm	No	No	Lucifer yellow (0.44 kDa), 4 and 70 kDa dextran, FITC-transferrin (78 kDa)	Membranes for lung-on-a-chip	[2]
PDMS	Porous, flexible membrane	10 µm	No	No	Fluorescently-labeled albumin	Membranes for lung-on-a-chip	[3]
Silicon carbide	Non-porous membranes	50 – 150 nm	No	No	N/A	Miniaturized cell culturing devices	[4]
Poly (4-hydroxy styrene)	Porous and flexible membranes	3 µm	No	No	Sodium fluorescein (376 Da)	*In vitro* models of BBB and intestinal barrier	[5]
Silicon dioxide	Ultrathin and porous membranes	300 nm	No	No	Indirect	Cellular barriers and co-culture models	[6]

PTFE, PET, polycarbonate	Porous membranes	40, 10, and 7 – 20 µm	Only the PTFE	No	20 kDa FITC-dextran	Membranes for a microfluidic model of the human airway	[7]
Poly-ε-caprolactone	Electrospun, nanofiber mesh	< 10 µm	Yes	Yes	Sodium fluorescein (376 Da)	Model of the alveolar-capillary barrier	[8]
Biologic polymers							
Collagen vitrigel	Non-porous membrane	20 - 50 µm	Yes	Yes	N/A	Corneal epithelial, stromal, and endothelial constructs	[9]
Collagen type I, Collagen type I + alginate, Collagen type I + Matrigel™	Fibrous and porous membranes	20 µm	Yes	Yes	20 kDa FITC-dextran	Membranes for microfluidic chips	[10]
Collagen type I, Matrigel™	Non-porous membranes	300 nm at the center and 1.3 µm at the edges	Yes	Yes	Indirect	Membranes for microfluidic chips	[11]
Gelatin/chitosan	Electrospun nanofibrous and porous membranes	11 – 14 µm	Yes	Yes	N/A	Substrate for modelling Bruch's membrane	[12]
Recombinant spider silk proteins	Non-porous membranes	3.4 – 9.3 µm	N/A	Yes	10, 70, and 500 kDa FITC-dextran	Substrate for modelling Bruch's membrane	[13]

Table S2. An inventory of primary antibodies against proteins of interest used in immunofluorescence.

Protein of interest	Primary antibody	Dilution factor	Manufacturer
Collagen I	Mouse anti-human	1:500	Abcam
Collagen III	Rabbit anti-human	1:500	Novus Biologicals
Elastin	Guinea pig anti-human	1:160	Abcam
Hyaluronic acid	Rabbit anti-human	1:200	VWR International
Zona-occludens 1	Mouse anti-human	1:200	Thermo Fischer Scientific

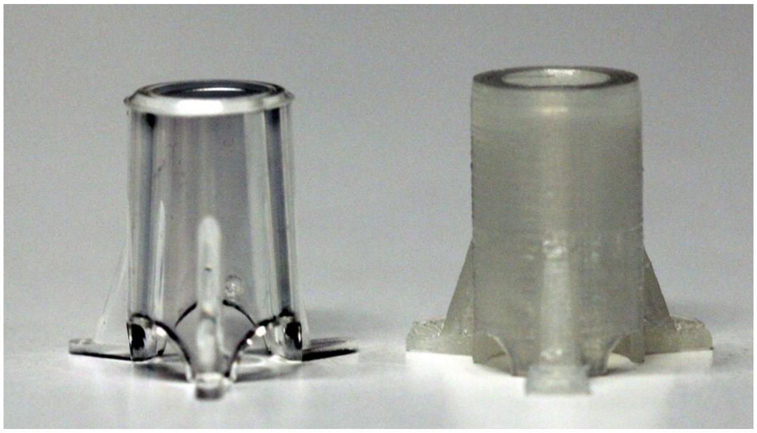

Figure S1. Photograph of a tissue culture insert (TC-insert, Sarstedt) (left) and a custom-made 3D printed holder used for capturing of silk membranes (right).

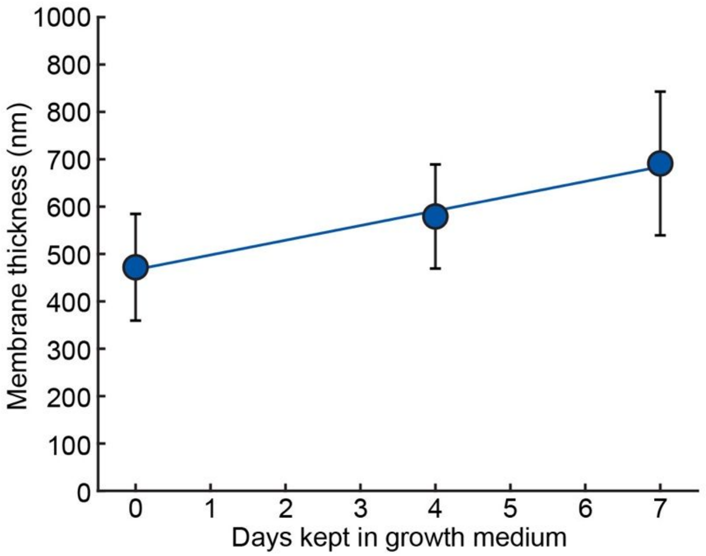

Figure S2. The thickness (mean ± SD) of silk membranes increases linearly with time due to adsorption of serum and growth factor components contained in the media on either side of the membranes (R^2 = 0.99).

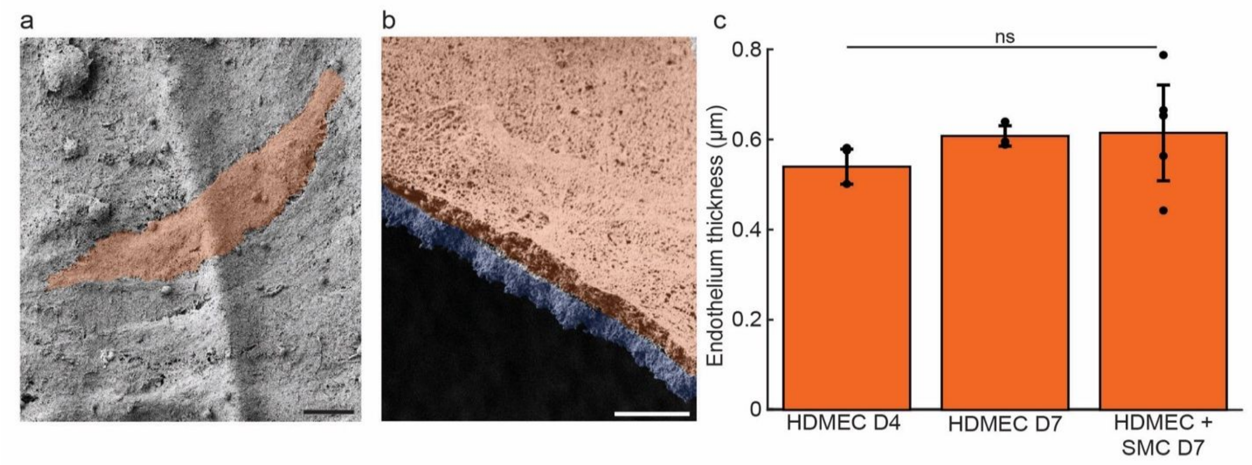

Figure S3. Scanning electron microscopy (SEM) images of a) flattened endothelial cells (HDMEC) on the air-side (apical) of a silk membrane after 4 days in culture, where one representative cell has been false coloured orange, and b) the air-side of a silk membrane (false coloured blue) with a confluent endothelium (false coloured orange) after 7 days in culture. Scale bars = 10 µm in a and 2 µm in b. c) the thickness (mean ± SD) of the endothelium on silk membranes under three different conditions: with only HDMEC at day 4, with only HDMEC at day 7, and with both HDMEC and smooth muscle cells (SMC) at day 7. ns - not significant (P > 0.05). HDMEC: human dermal microvascular endothelial cells; SMC: smooth muscle cells.

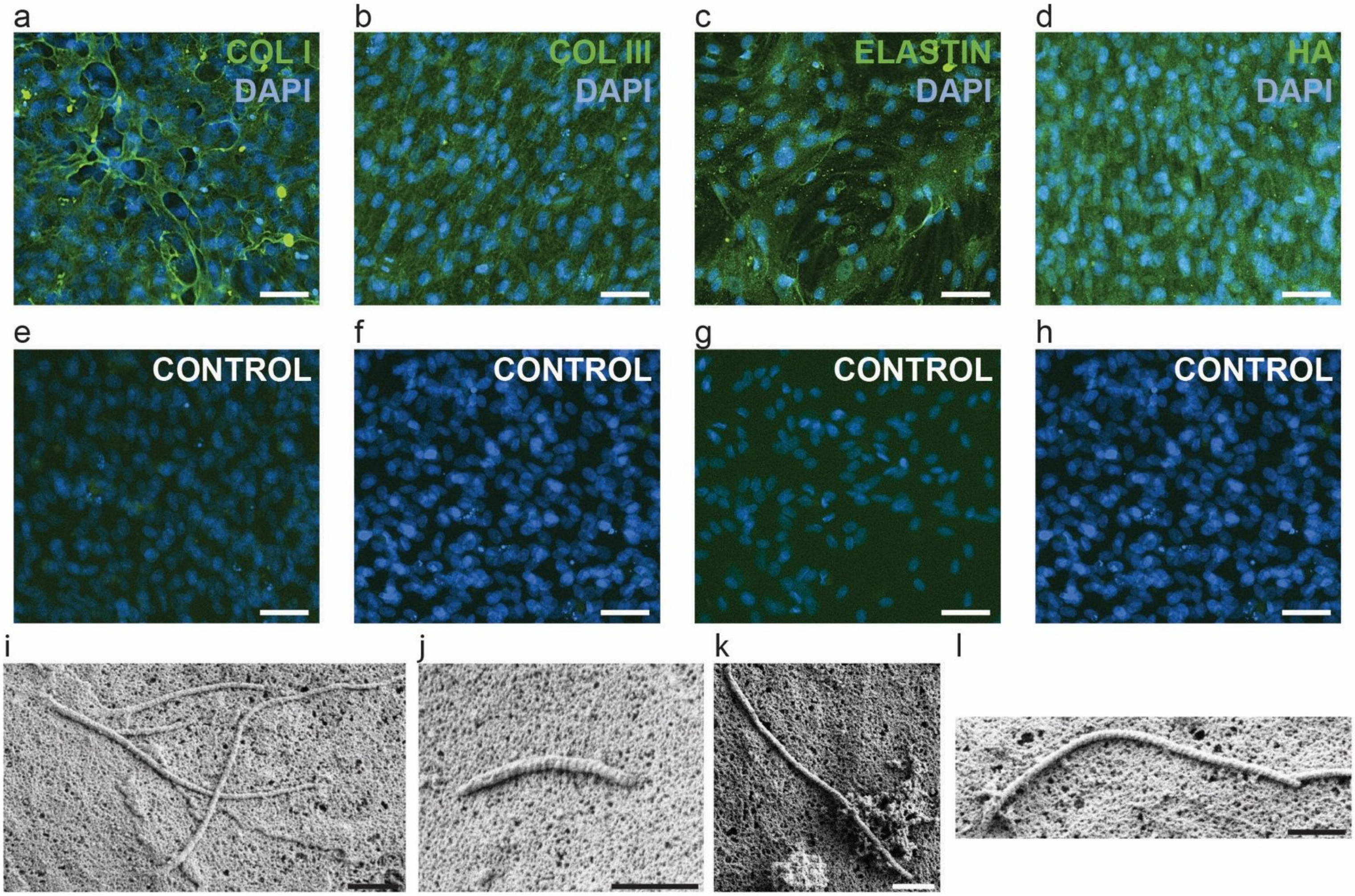

Figure S4. Key components of the extracellular matrix detected by immunofluorescence and scanning electron microscopy (SEM). Endothelial and smooth muscle cells on silk membranes after 7 days in culture stained for a) collagen type I, b) collagen type III, c) elastin, and d) hyaluronic acid (in green), and e-h) corresponding controls (excluding primary antibody) at 10x magnification. Cell nuclei stained by DAPI (in blue). Scale bars = 50 µm. Top-view SEM images showing the development of fibrillar structures on the air-side (apical) of i-j) double-seeded and k-l) seeded with only endothelial cells silk membranes. All at day 7. Scale bars = 1 µm.

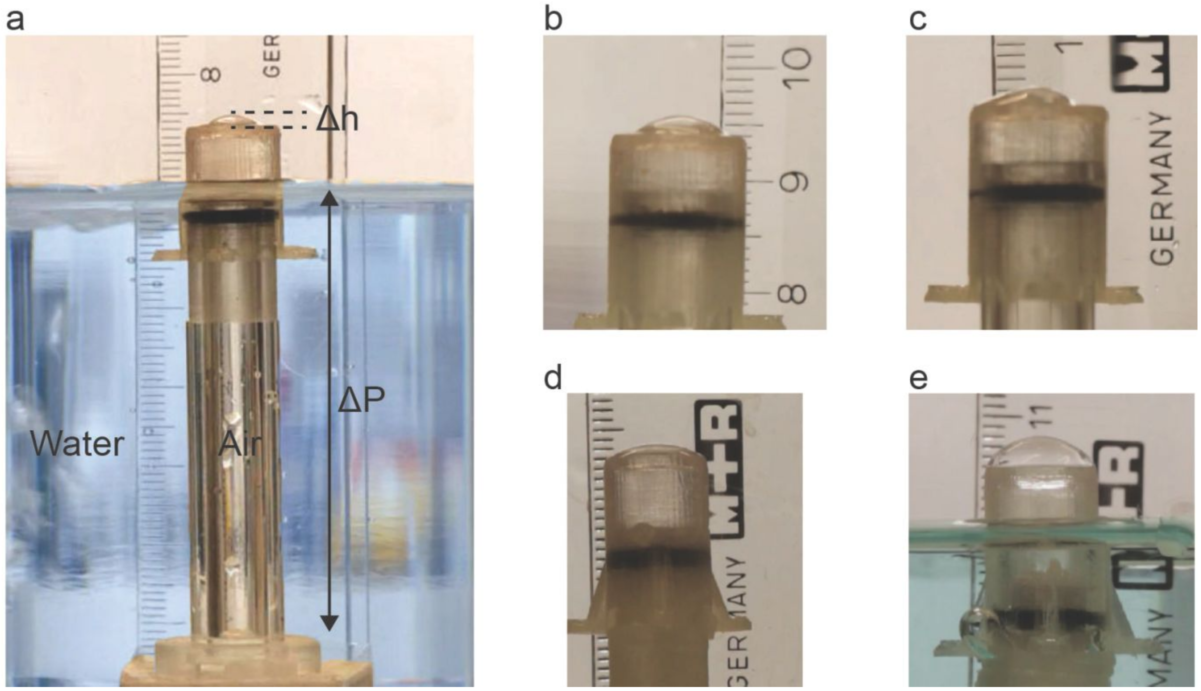

Figure S5. a) Photograph of the setup used for bulging experiments. Δh indicates the height that the membrane has bulged and ΔP the pressure difference inside and outside the membrane. Water outside the column has been false colored blue. The remaining photographs show four different bulging shapes, where b) central, small bulge, c) side-way bulge, d-e) wide bulges. Ruler is mm-scaled.

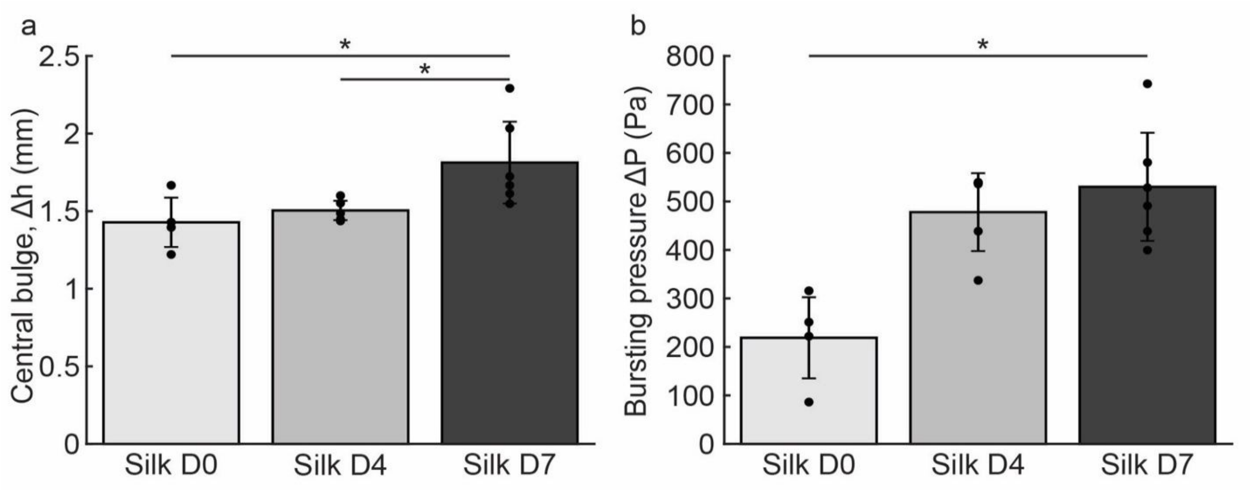

Figure S6. Mechanical properties of silk membranes without cells after 0, 4, and 7 days kept in cell culture media, where a) shows bulging (mean ± SD) and b) the corresponding pressure (mean ± SD) at burst. * $P < 0.05$

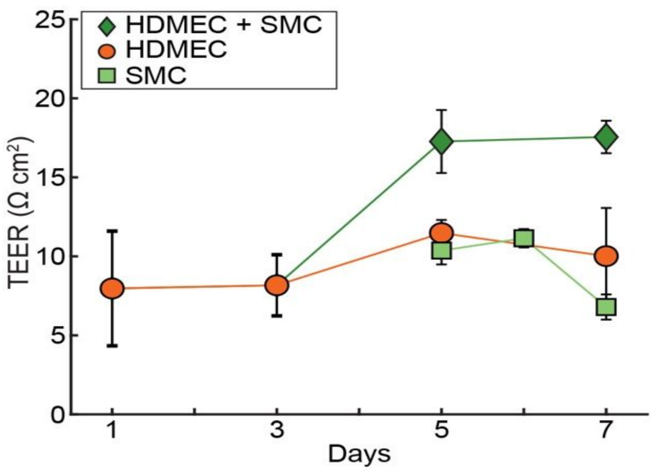

Figure S7. Normalized transendothelial electrical resistance (TEER) values (mean ± SD) for silk membranes seeded with both endothelial and smooth muscle cells (HDMEC + SMC), with only endothelial cells (HDMEC), and with only smooth muscle cells (SMC) at different time points. HDMEC: human dermal microvascular endothelial cells; SMC: smooth muscle cells.

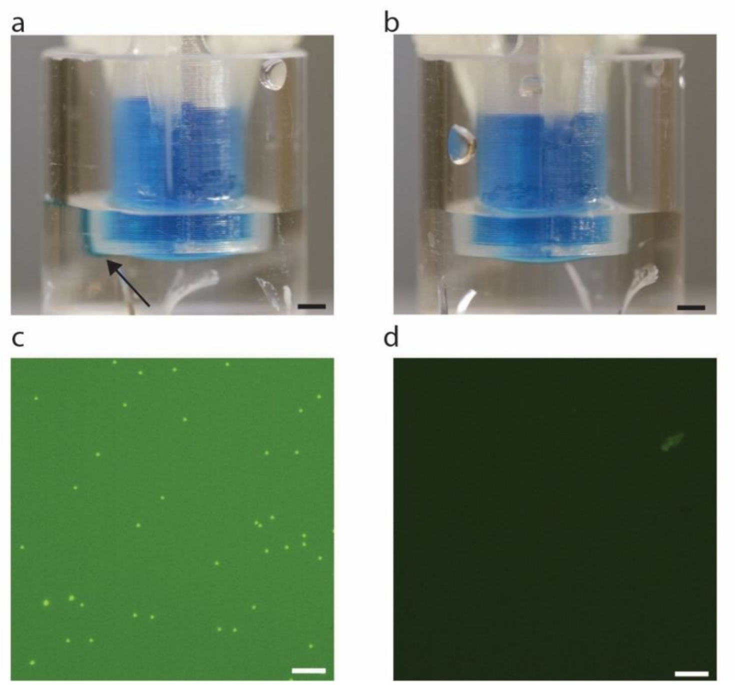

Figure S8. Photographs of a) a leaky silk membrane (black arrow points to the leakage point) and b) an intact silk membrane filled with diluted patent blue for observation purposes. Scale bars = 2 mm. Fluorescence images of the solution below c) a leaky and d) an intact silk membrane after completion of a permeability experiment. The microbeads observed in (c) are 3 µm in diameter. Scale bars = 100 µm.

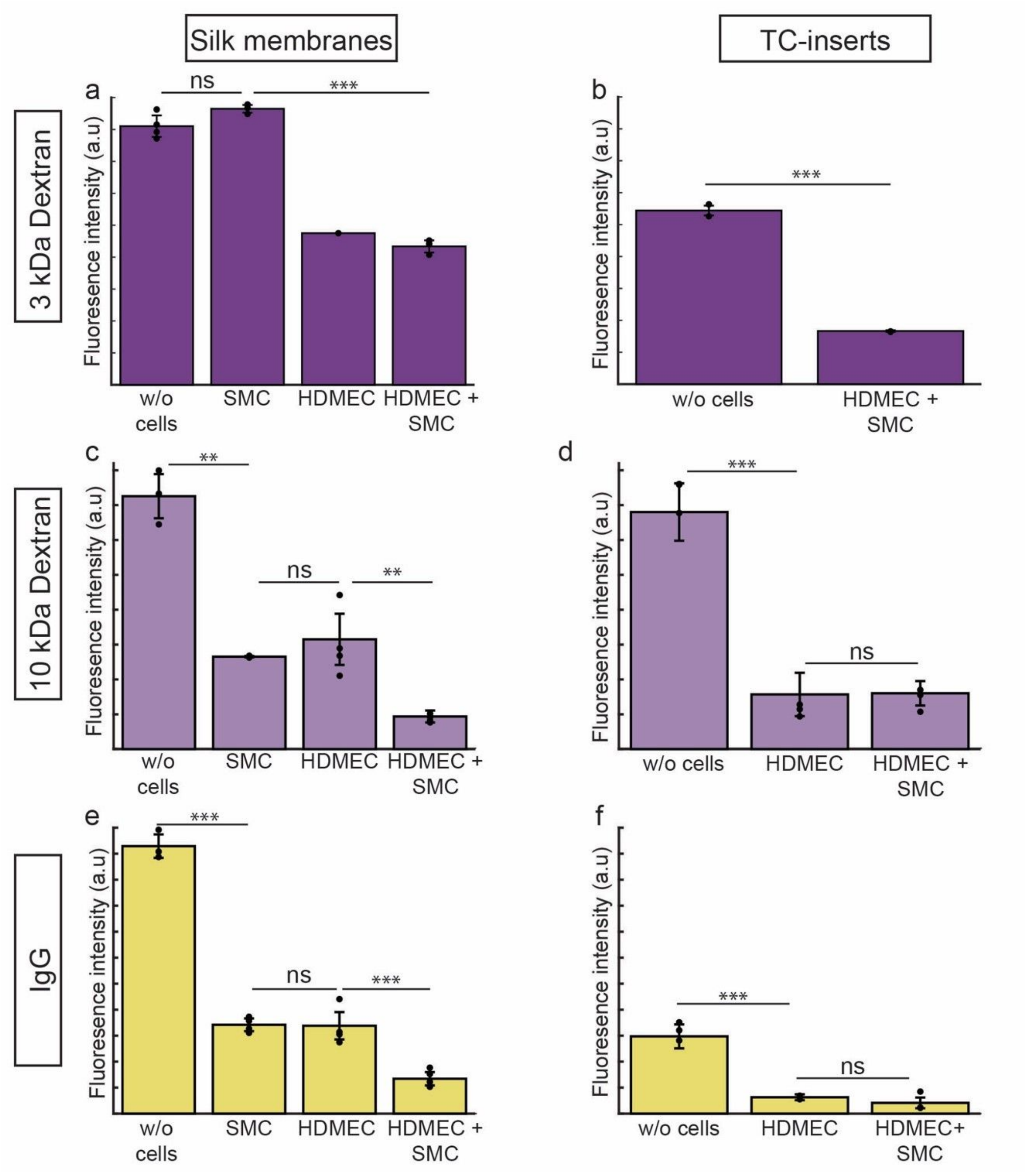

Figure S9. Permeation (mean ± SD) of a-b) 3 kDa Dextran, c-d) 10 kDa Dextran, and e-f) IgG for silk membranes (a, c, and e) and TC-inserts (b ,d, and f) without cells, seeded with only smooth muscle cells (SMC) (only for silk membranes), with only endothelial cells (HDEMC), and with both cell types (HDEMC + SMC). All after 7 days in culture, except for the silk membranes without cells for 3kDa dextran which were 4 days in culture. *** $P < 0.001$, ** $P < 0.01$, ns - not significant ($P > 0.05$). HDMEC: human dermal microvascular endothelial cells; SMC: smooth muecle cells.

References

1. Walter, F.R., Valkai, Sándor, Kincses, András, Petneházi, András, Czeller, Tamás, Veszelka, Szilvia, Ormos, Pál, Deli, Mária A., Dér, András., *A versatile lab-on-a-chip tool for modeling biological barriers.* Sensors and Actuators B: Chemical, 2016. **222**: p. 1209-1219.

2. Frost, T.S., et al., *Permeability of Epithelial/Endothelial Barriers in Transwells and Microfluidic Bilayer Devices.* Micromachines (Basel), 2019. **10**(8).

3. Huh, D., et al., *Reconstituting organ-level lung functions on a chip.* Science, 2010. **328**(5986): p. 1662-8.

4. Nguyen, T.K., et al., *Superior Robust Ultrathin Single-Crystalline Silicon Carbide Membrane as a Versatile Platform for Biological Applications.* ACS Appl Mater Interfaces, 2017. **9**(48): p. 41641-41647.

5. Shayan, G., et al., *Synthesis and characterization of high-throughput nanofabricated poly(4-hydroxy styrene) membranes for in vitro models of barrier tissue.* Tissue Eng Part C Methods, 2012. **18**(9): p. 667-76.

6. Carter, R.N., et al., *Ultrathin transparent membranes for cellular barrier and co-culture models.* Biofabrication, 2017. **9**(1): p. 015019.

7. Sellgren, K.L., et al., *A biomimetic multicellular model of the airways using primary human cells.* Lab Chip, 2014. **14**(17): p. 3349-58.

8. Dohle, E., et al., *Human Co- and Triple-Culture Model of the Alveolar-Capillary Barrier on a Basement Membrane Mimic.* Tissue Eng Part C Methods, 2018. **24**(9): p. 495-503.

9. McIntosh Ambrose, W., Salahuddin, Afrah, So, Stephen, Ng, Shengyong, Ponce Marquez, Sara, and T. Takezawa, Schein, Oliver, and Elisseeff Jennifer *Collagen Vitrigel Membranes for the In Vitro Reconstruction of Separate Corneal Epithelial, Stromal, and Endothelial Cell Layers.* Journal of Biomedical Materials Research Part B: Applied biomaterials, 2009. **90**(2): p. 818-831.

10. Mondrinos, M.J., et al., *Native extracellular matrix-derived semipermeable, optically transparent, and inexpensive membrane inserts for microfluidic cell culture.* Lab Chip, 2017. **17**(18): p. 3146-3158.

11. Iwadate, H., Yamada, M., Kimura, N., Hashimoto, R., Yajima, Y., Utoh, R., Seki, M., *PDMS microstencil plate-supported fabrication of ultra-thin, condensed ECM membranes for separated cell coculture on both surfaces.* Sensors and Actuators B: Chemical, 2019. **287**: p. 486-495.

12. Noorani, B., Tabandeh, F., Yazdian, F., Soheili, Z., Shakibaie, M., and Rahmani, S., *Thin natural gelatin/chitosan nanofibrous scaffolds for retinal pigment epithelium cells.* International Journal of Polymeric Materials and Polymeric Biomaterials, 2017. **67**(12): p. 754-763.

13. Thomas I. Harris, C.A.P., Farhad Farjood, Ian D. Wadsworth, Lori Caldwell, Randolph V. Lewis, Justin A. Jones, and Elizabeth Vargis, *Utilizing Recombinant Spider Silk Proteins To Develop a Synthetic Bruch's Membrane for Modeling the Retinal Pigment Epithelium.* ACS Biomaterials Science & Engineering, 2019. **5**(8): p. 4023-4036.
